# Air Quality Assessment in Pig Farming: The Italian Classyfarm

**DOI:** 10.3390/ani13142297

**Published:** 2023-07-13

**Authors:** Eleonora Buoio, Chiara Cialini, Annamaria Costa

**Affiliations:** Department of Veterinary Medicine and Animal Science (DIVAS), Università degli Studi di Milano, Via dell’Università 6, 26900 Lodi, Italy; eleonora.buoio@unimi.it (E.B.); chiara.cialini@unimi.it (C.C.)

**Keywords:** air quality assessment, animal welfare, pigs, aerial pollutants, pollutants measurements

## Abstract

**Simple Summary:**

Reducing the sources of stress on farms allows for enhanced animal welfare and productivity. Aerial contaminants and pollutants that can be found in indoor animal houses are among these stressors. In Italy, the guidelines to assess animal welfare in pig farming are displayed in a protocol named “ClassyFarm”, based on European legislation. Specific indications are given on the microclimatic conditions of livestock indoor environments (temperature, relative humidity, dustiness) and air quality, especially regarding harmful gases such as ammonia (NH_3_) and carbon dioxide (CO_2_). Nevertheless, the recommended measurement techniques for dust and harmful gases are not satisfactory. In this review, the effects of these pollutants that have been studied in the scientific literature are reported, as well as some tips that can help in carrying out proper measurements. The presence of harmful bioaerosol on farms cannot be neglected since it affects not only breeding animals’ well-being but also the health of people living in the surrounding area.

**Abstract:**

On 24 September 2019, the Ministry of Health issued an explanatory circular containing clarifications on the implementation methods of the National Improvement Plan for the application of Legislative Decree 122/2011. The Plan states that “In all farms where weaning or fattening pigs are raised and in breeding farms which wean piglets (excluding those for self-consumption), a risk assessment is carried out by the veterinarian on the basis of three levels: insufficient, room for improvement and optimal”. ClassyFarm, a risk assessment tool for livestock farming, is applied in Italy to evaluate the level of welfare and management of animals from a variety of points of view. Essentially, the categorization risk introduced by ClassyFarm in pig farming depended on the obligation stated by the EU in Decree 122/2011 to avoid tail docking in piglets and, at the same time, to reduce the stressor aspects able to induce aggressive behavior among pigs, improving the welfare and health status of animals. Since ClassyFarm evaluates many aspects of the management of animal farming, our aims in this review are to discuss the topic from an environmental point of view: (1) to frame the indications of ClassyFarm to make a farm risk assessment based on pigs’ welfare; (2) to review environmental quality assessment in pig farms, and its repercussions on animal health and welfare; (3) to describe the most used sampling techniques of air pollutants measurements.

## 1. Introduction

Nowadays, the concept of the welfare of livestock animals is of great importance in public opinion. Therefore, in recent years, the regulation of farm animals’ protection has often been questioned, leading to the need for new protocols able to implement breeding conditions in compliance with the national legislation, according to consumers’ perceptions and preferences [1].

The main purpose of animal welfare rules is to reduce stress, enhance animal welfare, and maintain or increase farm productivity. In intensive husbandry, animal housing should provide protection and a good environment in general, whose quality depends on parameters such as, essentially, building type and management. Welfare is the general state of good physical and mental balance in which the animal is in harmony with the breeding environment [2]. Diseases, parasites, and feed consumption are the most important factors in assessing the health of livestock animals. Nevertheless, the environment, such as temperature, air speed, relative humidity, gas, and dust concentrations in livestock houses, plays an important role in achieving good conditions for the growth and productivity of the reared animals.

Indoor animal houses can be rich in harmful contaminants that could threaten animal safety, causing discomfort and distress, thus reducing their welfare [3].

Several studies have been carried out on the harmful effects of these air agents on livestock workers, primarily associated with respiratory and cardiovascular diseases [4]. Given the similarities between swine and humans, these studies have led to detailed investigations of the toxicity of these contaminants in pigs. High concentrations of pollutants inside buildings can constantly expose animals to bad aerial conditions and serious health problems or production deficits [5].

So, good management and proper infrastructure in intensive systems are key factors in improving animal welfare [6]. Currently, one of the most important goals is to acquire a new understanding of the levels and properties of indoor concentrations and emissions of pollutants to define a better indication of environmental parameters [3].

Regarding swine production, no country has a complete definition of the optimal indoor environmental parameters in its legislation. There are reference limits to avoid dangerous pollutant concentrations based on the recommendations of the Scientific Veterinary Committee (1997). These limits are currently the only ones recognized by national laws, but considering the recently developed pollution reduction technologies and monitoring and warning systems, it is necessary to set new limits. However, having concentration threshold values does not help to control the interaction among different airborne pollutants, whose co-presence has a strong influence on the respiratory tract [7].

Until approximately 30 years ago, air quality assessment for livestock consisted of basic parameters such as temperature and relative humidity. Later, good management practices and control techniques for toxic gases, especially ammonia, were developed (for the safety of workers rather than animals); however, dust and everything associated with it have been underestimated, leading to little understanding of their true impact on human and animal health [3,4,5]. Dust not only has harmful effects by itself, but it can pair up and react with acid gases, resulting in the formation of the finest and most dangerous particulate matter, and it can also carry harmful substances [8].

In this paper, the relationship between pig welfare and environmental rearing conditions will be investigated, considering the aerial contaminants reported from the literature on pig farming and gathering all the evidence on the adverse effects caused by inadequate rearing conditions that have been highlighted so far.

This critical analysis stems from the reflection on the guidelines about environmental parameters provided by ClassyFarm, which is an Italian protocol for the evaluation of animal welfare in breeding promoted by the National Reference Centre for Animal Welfare (CReNBA, 2019).

## 2. The Introduction of ClassyFarm, the Italian Risk Assessment in Livestock Farming: The Swine Husbandry

In 2019, the Ministry of Health, with the support of the CReNBA (2019), developed new checklists available to official veterinarians with the aim of making the verification of animal welfare conditions easy, authoritative, homogeneous, and validated in Italian farms based on current regulations on the protection of farm animals and on the most recent and authoritative scientific studies.

The final aim of the application of the new protocol, in addition to allowing the identification of dangerous situations for animal welfare conditions, is to categorize the farms into “risk ranges” and make comparisons with the national, regional, and provincial realities.

The ClassyFarm system classifies farms according to three levels of risk:

− Level 1 = high risk, unacceptable/negative/dangerous condition, or stress, indicates the possibility that some of the animals are experiencing or may experience a negative situation (“distress”) due to the impossibility of fully enjoying one or more of the 5 freedoms;
− Level 2 = controlled risk or acceptable condition, compatible with the possibility that all herd animals can satisfy their five freedoms and are not subjected to stressful conditions;
− Level 3 = low risk or optimal, positive, and beneficial condition, due not only to full adaptation of the animal to its environment and respect for the five freedoms but also to the possibility of being able to live positive, fulfilling, and satisfying experiences capable of producing “eustress”.

The classification of risk level is addressed to preventive interventions on the main weaknesses of the rearing management system of each farm to improve the life conditions of the reared animals.

This procedure of animal welfare assessment takes into account the numerous indications provided by Legislative Decree 146/2001 on the protection of farm animals, the measures envisaged in Legislative Decree 122/2011 on the protection of pigs, the Circular of the Ministry of Health 0022766-P-12/12/2012 on the interpretative areas of Directive 2008/120/EC, and EU Recommendation 2016/336 relating to the application of Directive 2008/120/EC of the Council. This directive establishes the minimum standards for the protection of pigs in relation to measures aimed at reducing the need for tail docking in the reports and scientific publications of the most important research groups and European bodies, including the European Food Safety Authority (EFSA).

The method is based on the analysis of two groups of data: those connected to the dangers derived from environmental conditions (management, structures, equipment, and microclimatic conditions) and those derived from the survey of the most important direct indicators of well-being or animal-based measures (ABMs) provided by the most recent scientific literature.

The main challenge encountered was the almost non-existence of chronic exposure experiments, reflecting farm reality. However, several studies on acute toxicity exist, but the values investigated are far from those present in farms. These tests aimed to establish exposure limits for swine in the past [4], but they do not reflect the actual farm situation.

Another problem was the lack of interdisciplinary studies; in most cases, the studies deal only with one or a few variables and do not consider the interaction between them. Since air quality depends on different factors, each of them has an individual effect as well as a synergic one, and it is important to consider all of them. What seems to be missing, probably due to the many variables to consider, is a comprehensive study of the whole production cycle through the investigation of behaviors, injuries, health status, and animal performance.

There are two principal protocols for swine welfare assessment: the welfare quality assessment protocol, applicable in the European Union, and the swine welfare assurance program, applicable in the USA. The first is mostly focused on animal-based measures, and the second on environment- and management-based measures [9].

In Italy, ClassyFarm is an integrated system aimed at categorizing farms according to risk, considering the relationship between animal health and public health, the environment, animal welfare, antimicrobial resistance, and food security. To do this, the parameters of the welfare quality and the documents by the European Food Safety Authority (EFSA) were combined to facilitate the introduction of a real risk assessment based on animal welfare.

The latter is defined by four principles: appropriate housing, proper feeding, good health, and welfare. These principles are expressed through 12 welfare criteria that, in turn, are applied through 30 measures.

Thus, animal welfare has become part of the One Health concept, considering its influence on people and the environment. The system is based on the analysis of two groups of data: those provided by Legislative Decrees 146/2001 and 122/2011 related to environmental conditions (management, structures, equipment, and microclimatic conditions) and those derived from direct animal-based indicators provided by the latest scientific literature.

ClassyFarm enables data collection and processing in the following assessment areas:

- Area A—Personal and corporate management;
- Area B—Facilities and equipment;
- Area C—Animal-Based Measures (ABMs);
- Area biosafety and biosecurity;
- Great risk and alarm systems.

Regarding environmental conditions, this system is based on European legislation (type of flooring, lighting, and noise regulation) and measures used in classic welfare determination systems. To establish animal responses to breeding conditions, major attention is often given to animal-based measures (ABMs). Nevertheless, ABMs are not good early warning indicators for assessing poor air quality. The reassuring aspect is that most animals, except for individuals with individual sensitivities, should be able to recover their status quickly if environmental conditions are corrected early enough. The final thought is about the assessment of farm animal welfare. The authority for welfare control in livestock farming is the veterinarian, who interfaces between the operator and the law and suggests strategic farm management choices to improve animal health and welfare conditions based on the risk level detected. The parameters observed are mostly animal-based, but the check of microclimatic conditions includes many aspects related to animal welfare that need specific transversal knowledge across animal science and bioengineering in livestock systems, allowing us to overcome classical concerns and emphasizing the role of air quality in determining a good welfare status in livestock animals.

In area B of ClassyFarm, specific indications are given on the microclimatic conditions of livestock indoor environments, where the sections concerning microclimate parameters and air quality are organized as follows:

- Temperature, relative humidity, and dustiness (B 36);
- Harmful gases: ammonia (NH_3_) and carbon dioxide (CO_2_) (B 37).

In this paper, the parameters that define the microclimatic conditions (temperature, humidity, and air velocity) were examined first, followed by air pollutants, such as harmful gases and dust concentration.

## 3. Microclimatic Parameters

In Italian piggeries, most animals are raised for a period of approximately nine months (corresponding to 160 kg of Live Weight, LW) to produce fattened pigs for Parma or San Daniele ham production, typical made in Italy cured hams.

During this period, chronic exposure to environmental stressors such as aerial contaminants in confined houses can cause health and production problems [10].

Ni et al. [3] in their review highlighted that air quality began to receive attention in the 1930s [11], finding great insights from the 1960s to the 1980s, and then losing importance until 2010. Fortunately, recent advances have led to new research aimed at understanding dust composition, what it can transport, and how particulate matter interacts with gases such as NH_3_. All of these long-ignored concepts have had a powerful impact on the welfare and health of both animals and humans.

Generally, in swine confinement, several aspects must be considered to provide adequate environmental parameters. The microclimatic parameters (temperature, relative humidity, and air velocity) are essential elements of the indoor environment.

− The temperature is the average air temperature registered in the room. It is expressed according to the Celsius scale in degrees Celsius (°C);
− Relative humidity is the ratio, expressed as a percentage, between the actual amount of water vapor contained in the air at a given temperature (absolute humidity) and the maximum amount possible (saturated vapor) at the same temperature;
− Air velocity is the ratio of the distance covered by air flow, referred to as time t, and is expressed in meters per second (m/s);
− Although not explicitly discussed in the ClassyFarm system, the heat losses occurring in an animal environment through conduction, convection, radiation, and evaporation may be taken into account during environmental monitoring sessions since the surface thermal insulance of the surface-air boundary layer depends on radiant and convective conditions.

Temperature is the easiest parameter to control. In the ClassyFarm system, the optimum temperature value was provided based on the literature related to animal weight (Table 1).

Table 2 shows the temperature levels identified for rearing pigs of different weights and under different housing conditions.

Evaluating the internal temperature of pig farms needs to consider the type of building design since it can affect the thermal response of animals. In Table 2, the Lower Critical Temperature (LCT) for pigs of different weights in different housing conditions is reported.

**Figure 1:**
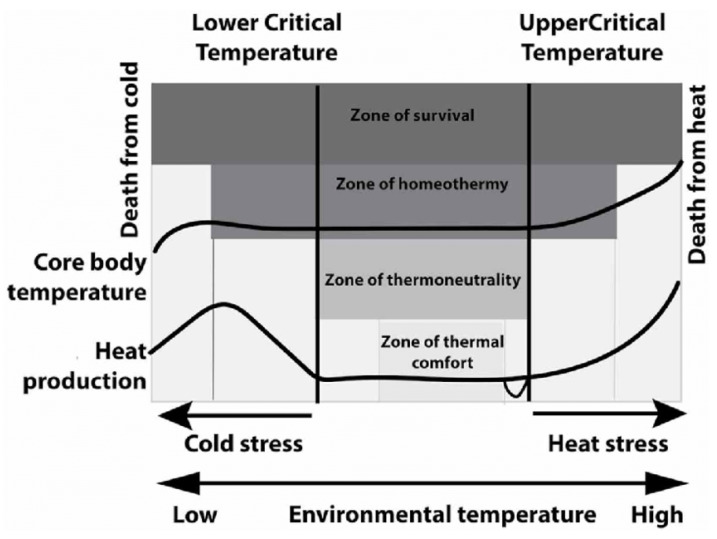
The concept of the thermoneutral zone (Lower critical temperature—LCT; Upper critical temperature—UCT) modified from [14].

However, a wide body of literature reports that optimal temperature levels are combinations of animal weight, age, breed, physiological condition, dietary level, housing solutions, and environmental factors. Therefore, each animal has its own temperature comfort zone, delimited by the lower and upper critical temperatures beyond which the organism must expend additional energy to maintain homeothermy in the thermoneutral zone (see Figure 1).

High-performance animals experience high metabolic heat production owing to increased metabolic rates. In addition, to ensure a higher standard of animal welfare, the temperature level must be adjusted based on other factors such as relative humidity, animal density, season, type of flooring, and type/efficiency of the ventilation system.

The occurrence of high and low ambient temperatures, increased frequency of disease [15], and increased mortality due to immunosuppression or increased pathogen pressure may lead to negative effects on fertility and other performance traits, such as weight gain and feed conversion rate. Pigs are very sensitive to high ambient temperatures and can modify their behavior to protect themselves. At hot temperatures, pigs adopt a side-lying position to provide the maximum surface area and the widest contact with the ground and seek isolated positions away from others, possibly in areas of stronger airflow and reduced activity levels. In addition, they restrict food intake but significantly increase drinking water consumption. Pigs are also used to getting wet with water or feces to feel fresher. In breeding pigs, temperatures above 32 °C can lead to reproductive problems in both the sows and boars; in lactating sows, high temperatures limit feed intake and reduce milk production [13].

Concerning the relative humidity, the indicated optimal range is 50–75% [16]. Low relative humidity can cause problems in the respiratory tract due to dehydration of the mucous membranes and an increase in dust in the environment. High humidity can be extremely damaging, particularly when combined with high or low temperatures. High relative humidity and low temperatures contribute to body heat loss, whereas the opposite makes thermoregulation difficult [3,4,5]. Instead, low humidity causes energy waste and increases dust in the environment, and relative humidity is the first index that, in winter, provides an indication of correct ventilation [3,4,5].

Regarding air speed, whose recommended ranges are 0.2 to 3 m s^−1^ in piggeries [12] according to the housing solution, the optimal value varies according to the season because its effects are closely related to temperature. In fact, in winter, a lower velocity ensures limited heat loss from the animal’s body. In summer, however, it can be useful because, by accelerating skin evaporation and heat loss, thermal stress is effectively limited.

Because temperature and humidity can be easily regulated through controlled systems, air quality is often sacrificed to ensure specific thermal conditions and maximize production performance [17]. For example, during the winter period, to preserve the temperature and humidity, the ventilation rate is reduced, causing a lower air exchange that leads to a higher concentration of pollutants. The same occurs during the night, when the ventilation rate is usually reduced to limit energy consumption [18].

## 4. Pollutants Generated in Piggeries: Gasses and Particulate Matter

In the UE and in the Italian legislation (D.L. 146/2001, all. 1, par. 10), the indication given about livestock air quality is to keep “the air circulation, the amount of dust, the temperature, the relative humidity, and the gas concentration within certain limits that are not harmful to animals”, but no limits are given. As mentioned above, defined maximum limits were provided by the Scientific Veterinary Committee (1997, Table 3).

Currently, different welfare protocols are available, but they are voluntary-certification systems whose aim is to make the farm more environmentally and animal-friendly.

Air pollutants generated by swine husbandry include gases such as ammonia, carbon dioxide, nitrous oxide, methane, and hydrogen sulfide, but also particulate matter and microbial by-products. Methane, in piggeries, is originated by anaerobic fermentation of slurry at high levels in deep pits, and it can be considered nontoxic. [4,19].

Pigs raised in inadequate air quality conditions have a lower feed intake, a worse feed conversion efficiency, and an increased cortisol concentration [20]. In addition, in polluted environments, animals may show less playful behavior [21] and be more prone to aggressivity and cannibalism phenomena [8,22], resulting from a sense of disorder.

Table 4 shows the possible outcomes of different pollutants at low concentrations on animal health.

There are several experimental studies on the effects of these gases on animal health, as well as recent reviews [3,4,23,24]. Experience shows that high concentrations of harmful gases mainly occur in housing systems with insufficient ventilation and high temperatures, while open environments have higher air circulation and do not present such problems.

In ClassyFarm, specific limits are present only for ammonia and carbon dioxide, the indicated reference for an appropriate condition of NH_3_ and CO_2_ must be 10–20 ppm and less than 3000 ppm, respectively; an NH_3_ concentration lower than 10 ppm is considered the acceptable one.

### 4.1. Carbon Dioxide

Carbon dioxide is a greenhouse gas and derives mainly from the respiration of animals that inhale air containing 0.035% CO_2_ and exhale it containing 5% CO_2_. Animal production of CO_2_ depends on the species, body weight, and diet. To a lesser extent, CO_2_ production is due to the degradation of organic substances [25], such as those contained in manure. Indeed, based on manure management and the type of barn, different concentrations can be found. In particular, on slatted floors with deep pits in which slurry is stored for a long period of time, the CO_2_ concentration can be up to 10% higher. Also, litter presence can contribute to CO_2_ production. Litter emissions are more difficult to calculate; however, it has been shown that bedding can produce the same quantities as animal CO_2_ production [23,26].

As indicated in ClassyFarm, the acceptable level of carbon dioxide in animal houses is below 3000 ppm [25], although in winter, with a minimal ventilation rate, it can reach 6000 ppm [3]. Its concentration strongly depends on the ventilation rate, and for this reason, there is an important diurnal variation to consider during CO_2_ measurements. Mainly during the night, with a reduction of the ventilation rate, the concentration of CO_2_ in animal husbandry can increase by about 20% [23].

At high concentrations, animals experience dizziness and unconsciousness, while at very high concentrations (30,000 ppm), it can cause death by asphyxiation.

One characteristic of CO_2_ is that, by being heavier than air, it tends to stratify downward. Therefore, during environmental measurements for its detection, it would be better to measure its concentrations at different heights.

### 4.2. Ammonia

Ammonia is the most studied livestock pollutant. It comes from the biological degradation of nitrogenous organic substances (urea and uric acid) contained in urine and feces [8]. It is a colorless gas that can cause a systemic inflammatory response [27].

Usually, in swine confinement, NH_3_ is present in different concentrations, as shown in Table 5.

In ClassyFarm, a restrictive range for ammonia is reported: 10 to 20 ppm. The first value is the maximum limit accepted for prolonged exposure in humans.

In most countries, the threshold limit concentration in piggeries is 25 ppm; as regards human health, the current recommended maximum exposure standards vary from 7 to 25 ppm depending on the country and the exposure time, short-term, 15 min, or workday, 8–10 h [5,8,28].

Preference studies have been conducted to understand whether pigs are able to discriminate a polluted place from a cleaner one, but the flaw in these studies is the pollutants concentration. For example, in Drummond [29], NH_3_ concentrations of 50, 100, and 150 ppm were tested, but those levels do not reflect real farm conditions. In fact, as indicated by De Boer and Morrison [30], the ammonia concentration in piggeries generally ranges from 1 ppm to 30 ppm.

In another study, the preference test was performed at 5–10 ppm versus 100 ppm, finding that animals have different tolerances to aerial ammonia. It was also noted that the choice of laying area is strongly related to the animal social hierarchy [10]. The first threshold (5–10 ppm) was chosen because these limits are not toxic for animals. However, we know from Jones [31] that 10 ppm is enough to create discomfort in pigs, and these values are also bothersome to humans. Exposure to ammonia, alone or in combination with other pollutants, has detrimental effects on animals, such as eye inflammation, coughing, sneezing, and dyspnea, depending on the duration of exposure and the amount of pollutant. It is a very dangerous gas, especially for young pigs, whereas in adults there may be less damage due to the animal’s major resistance.

As reported by Gustafsson [24], high NH_3_ concentrations have an important influence on animal growth performance since feed intake and nutrient utilization efficiency are reduced. This could also depend on changes in gut integrity that concern gut morphology, intestinal bacterial flora, metabolites, and gene networks [32]. However, a compensatory response has been shown following long-term ammonia exposure: while food intake decreased, food conversion efficiency increased [33]. This may indicate that animals have to work harder to deal with the environment and, as a result, may be an indicator of potentially compromised welfare. In Table 5, some ammonia values recorded in piggeries are reported.

**Table 5:** Some values of ammonia concentration measured in swine buildings.

Year of the Study	Reference	Ammonia Concentration (mg/m^3^)
1974	[34]	18
1980	[35]	0.01–1.9
1981	[36]	2.8-15.3
1982	[37]	0.1–18
1982	[38]	1–24
1991	[39]	1.06–9.37
1996	[40]	3.65
1997	[41]	7.08–31.86
1997	[42]	14.66
1998	[28]	5–18 ppm
1999	[43]	12–30
1999	[44]	9 ± 1–15 ± 9 ppm
2000	[45]	7–15 ppm
2000	[46]	2.8–10.6 ppm.
2000	[47]	13.88
2003	[48]	Less than 30 ppm
2005	[49]	1.2–37 ppm.
2017	[19]	5.31–7.45
2022	[50]	2.14–5.71

All organisms exposed to NH_3_ undergo oxidative stress, which can cause a series of pathological damages to cells, inducing cell necroptosis and having further negative effects on animal immunity [8]. The high presence of oxidant radicals results in animals’ higher susceptibility to pathogens and diseases.

Moreover, the possible modification of epithelia due to the chronic inflammatory state may reduce the ability to absorb nutrients, resulting in a higher frequency of diarrhea.

In Li et al. [51], the effect of 90 mg of NH_3_ was studied, showing damage to the gut-brain axis through the induction of cell apoptosis. Moreover, some recent research has reported that ammonia exposure can interfere with lipid metabolism [52].

Another important aspect concerns the effects on the respiratory system.

Ammonia exposure can lead to respiratory symptoms such as coughing, rhinitis, and even pneumonia. Damage to the respiratory system may involve the upper airways and, under critical conditions and in the presence of fine particulate matter, may reach the lower compartment. The main symptom is mucus overproduction caused by nasal mucosa hyperplasia [27].

A critical respiratory condition involves the olfactory perception; the ability to recognize scents may be affected, with consequences such as feed palatability reduction [53]. Furthermore, it can have negative effects on pheromone reception and therefore on animal reproduction [22,54].

Recently, the effect on the liver has also been investigated since it is responsible for the detoxification activity of the organism. It has been shown that an excess of NH_3_ (approximately 90 ppm) can change the transcriptional profiles of pig liver, with consequent liver pathology and immune dysfunction [55].

Among many studies, NH_3_ toxicity on (in vitro) porcine oocytes was investigated to examine the negative implications in the reproduction sphere. The data suggest that factors such as actin disruption, ROS generation, early apoptosis, and autophagy could influence oocyte maturation [56]. Although it is an in vitro study, it could provide crucial information related to farrowing room biosafety.

### 4.3. Hydrogen Sulfide

In ClassyFarm, hydrogen sulfide is not reported among the noxious gases, probably because it results from the anaerobic digestion of slurry, it binds with water, and the greatest production comes from slurry agitation [4].

Nevertheless, hydrogen sulfide (H_2_S) can be very dangerous. It can cause inflammation of the respiratory system, irritation of the mucous membranes, reduced appetite, paralysis of the diaphragm, and be lethal at high concentrations [3]. The target organ is the central nervous system, so after acute exposure, animals can appear unconscious and in respiratory failure [4].

Reported incidents of poisoning or death are related to the closeness of slurry storage tanks during slurry agitation, from which high concentrations of H_2_S were released into the air [57]. So, in pig facilities, H_2_S is typically under the detection limit (approx. 1 ppm), but it can reach higher concentrations unless manure management is conducted in the correct way, especially when it comes to livestock with slatted floors and deep pits.

### 4.4. Particulate Matter

In animal buildings, dust can originate from both inorganic and organic sources, principally from feed [58] and bedding materials and less from animals (skin flaking, hair) and manure [50,59,60,61,62].

However, as well as ammonia, chronic exposure to high dust concentrations can lead to the development of diseases such as bronchitis, asthma, and chronic coughing due to its mechanically irritating effect. Therefore, further information about dust needs to be investigated.

Airborne dust represents a big risk both to farm operators and animals. Moreover, it can also pollute the surrounding area, increasing the risk of asthma and lung disease in nearby residents [63,64,65]. Particulate matter consists of coarse (PM _2.5–10_), fine (PM _1–2.5_), and ultrafine (PM < 1) particles.

Dust can be classified according to the aerodynamic size of the particles. Total Suspended Particles includes all the particle size fractions. Inhalable dust refers to the smallest airborne particles that can be breathed in, and it includes particles with an aerodynamic size up to 100 μm. Respirable dust is the larger particle that can move into the gas exchange lung area [66] and can carry harmful gases and microorganisms into the respiratory tract [67]. These particles can reach the lung area in an inversely proportional way to their size, since the smaller the particle diameter, the deeper the particles are deposited in the respiratory tract through different mechanisms with different negative effects: impaction, sedimentation, interception, and diffusion [22,61].

So, dust can be distinguished first for its particle size and morphology; it is determinant to know the transmission distance, the suspension time, and the location of deposition in the respiratory tract [65]. PM_10_ are particles smaller than 10 µm and they include dust, pollen, and spores. In contrast, PM_2.5_ refers to particles smaller than 2.5 µm, such as combustion particles, organic compounds, and metals.

In the ClassyFarm system, the topic of dust concentration is not examined in depth. They define an unsuitable condition as one in which dust does not allow one to see “the end of the shed”. Dust concentration is assessed on a black A4 sheet positioned at a height above the pigs and away from the feed dispensers. At the end of the evaluation, the dustiness level is assessed according to the amount of dust present (“none, little, a thin coating, a lot of dust, the color of the sheet is not recognizable”). The housing conditions are considered unsuitable when the color of the sheet is no longer recognizable.

This technique, proposed as a risk assessment for air pollution by dust in ClassyFarm, can present critical issues since the A4 sheet will be covered by Total Suspended Particles (TSP), which includes all particle size fractions, while the investigation may be directed to the finest fraction of dust, or at least to particles with an aerodynamic size less than 10 µm, using dust samplers, as reported in Table 6. The monitoring of fine particles could reveal potential high concentrations of dust having the capacity to penetrate deeply into the respiratory tract, triggering negative synergistic health effects in the lungs [8]. Moreover, particles with an aerodynamic size lower than 100 nm are captured by the blood stream in the zone of gas exchange in the alveoli, escaping the phagocytosis exerted by alveolar macrophages and thus initiating the formation of ROS [65], with adverse effects on animals and workers.

As reported in Table 6, particulate matter can be characterized under physical, chemical, and microbiological parameters.

Regarding the chemical composition, it is possible to investigate the particles’ origin (mineral or organic) and the potential presence of toxins and allergens. It is important to investigate not only the chemical composition of dust but also its morphological and aerodynamical characteristics in order to define the PM sources and their properties [61].

In the end, microorganisms contained in dust derive principally from animals’ excreta. This mixture forms the bioaerosol, which includes mostly bacteria and, to a lesser extent, viruses, fungi, and endotoxins. Moreover, ammonia and odors can bond with dust, and this is a problem considering that microorganisms can use ammonia as a source of nitrogen for their growth.

In addition, ultrafine particles (<1 μm) reacting with acidic gases, such as ammonium and nitrate sulfate, contribute to the formation of secondary particulates and may pose an additional health risk [65].

Pikridas [71] reported that relative humidity and ammonia play a fundamental role in the formation of new particles. Starting from ultrafine particles, through phase transformation, it is possible to obtain new and different ultrafine particles subjected to continual changes [8]. These molecules, being very small in size, can cause serious health damage.

Ammonia levels in dust can reach 0.9–7.2 μg mg^−1^ [72].

The threshold limit for organic dust varies; in many countries, the limit is 10 mg m^−3^ for total dust; in Denmark, it is only 3 mg m^−3^, although in barns that limit is often crossed. Wathes [70] indicated that 5 mg m^−3^ of dust can dramatically affect pigs’ performance, suggesting that the Danish threshold is probably more reliable.

In recent years, greater attention has been given to the evaluation of farm dust and airborne particles’ content and the possible risk to animals reared in enclosed buildings. The most important information is related to the difference in bonding compounds depending on the particulate dimension.

The hazardousness of PM is defined by its aerodynamic dimension: PM_2.5_ can directly reach the alveoli and the blood circulation, which makes it the most dangerous compound [65]. In swine confinement buildings, PM_2.5_ is the most prevalent [52].

High hygiene standards can help keep airborne particulate matter under control, but no limit regulation is provided for dust [7]. However, even if a limited value were given, it would not be enough because a deeper analysis of dust components is needed to understand its role in affecting animals.

Unlike other studies, Done et al. [66] found no statistical relation between air quality and clinical observations or animal performance. The authors realized a single, multifactorial study on ammonia and dust effects on weaned pigs, using nine hundred and sixty animals exposed for 5 weeks to different mean ammonia (0.6, 10.0, 18.8, or 37.0 ppm) and mean inhalable dust (1.2, 2.7, 5.1, or 9.9 mg m^−3^) concentrations to represent air quality in a commercial husbandry.

On the other hand, these results disagree with other studies, in which the exposure limits for inhalable dust and respirable dust were set at 3.7 mg/m^3^ (2.4 mg/m^3^ per human) and 0.23 mg/m^3^ [73,74].

As reported by several studies, high levels of NH_3_ and dust can increase the incidence of multifactorial respiratory diseases [66,74,75,76].

As said before, dust can contain dangerous substances for animal and human health, like gases, bacteria, fungi, viruses, and active endotoxins [61,77,78].

Endotoxins are implicated in hypersensitive pneumonia in humans and can also affect the immune system; they are sufficient to trigger an immune response and lead to a respiratory disease [77]. The levels of endotoxins can clearly represent a great hazard, both for workers and animals.

Seedorf [7] proposed a Livestock Burden Index (LBI) for airborne contaminants, to establish index classes indicating the magnitude of the burden to which the animals are exposed:

He was one of the first researchers to understand the necessity of defining the synergic effect between them. He defined a full equation to estimate LBI for pigs, in which he included ammonia (NH_3_), inhalable dust (ID), respirable dust (RD), and inhalable endotoxins (IEtox). The limit concentration was defined by the literature review.

Thresholds over which diseases and production problems can be developed are:

− An amount of 10 ppm of ammonia, which is enough to create animal discomfort [31];
− An amount of 3.7 mg m^−3^ of inhalable dust, 0.23 mg m^−3^ of respirable dust and 1.540 EU m^−3^ of endotoxins, that means 154 ng m^−3^ [79].

The equation of LBI for pigs is reported as follows in Equation (1):

(1) $$\mathrm{LBIp}=\frac{\mathrm{CNH}3}{10\;\mathrm{ppm}}+\frac{\mathrm{CID}}{3.7\;\mathrm{mg}\;m^{-3}}+\frac{\mathrm{CRD}}{0.23\;\mathrm{mg}\;m^{-3}}+\frac{\mathrm{CIEtox}}{154\;\mathrm{ng}\;m^{-3}}$$

where C is the measured concentration related to the defined TLV (Threshold Limit Value, intended as the environmental concentrations of airborne substances below which workers can be repeatedly exposed for a working life without any adverse health effects) of a specific component.

Dust particles are affected by different forces that define their diffusion and deposition in animals’ rooms. According to Cambra-Lopez [61], PM sources can vary among different swine facilities. Most PM originates from manure; its contribution is 70–98% in fine particles and 41–94% in coarse particles.

The defecatory habits, the building structure, and the manure removal system can affect the presence of airborne bacteria in PM, with different percentages in summer and winter, 59.4% and 19.9%, respectively [64]. This difference can be due to several reasons: firstly, the different temperature; secondly, the practice of emptying the pit; and also parameters such as RH and ventilation. The potential of fattening pigs to release manure-derived particles is three times greater than that of sows and piglets and may depend on the metabolism and maturity of the digestive system, as well as the feeding ratio [65,79].

In addition, particulate matter can be rich in antimicrobial resistance genes (ARGs), known as resistome. Luiken [80] found out that about 63–73% of dust resistome derives from the aerosolization of animal faces. However, it is clear that the other part of the resistome is defined by bacteria from other body parts and substrates present in the farm (feed, soil, and instruments).

Since antimicrobial resistance is part of the One Health concept, it is necessary to investigate this aspect of farm reality in order to understand the transmission risk.

Recently, Van Gompel [81] used a metagenomic shotgun approach to investigate the relationship between pig resistance and risk factors at the farm level, across nine European countries. Results show a positive correspondence between antibiotics used in association with macrolides and tetracycline resistance. On the other hand, no association was found for β-lactam resistance.

This aspect is very important because it provides some new information about fecal ARGs that can affect animals and operators, causing problems with antibiotic resistance.

The greatest worry concerns the public health risk because the farm airborne resistome represents a problem not only for farm workers but also for the community living near the facility [82].

Cui et al. [83] established that the abundancy and the variability of the bacterial population in dust increase according to pigs age, so that at the beginning and at the end of the breeding cycle there are different risk conditions.

The microbic concentration in dust has seasonal variations; it has much more diversity during summer and autumn, strongly influenced by temperature and humidity but not by PM_2.5_ concentration [52]. The easiest explanation is the relation between the growth and survival of certain taxa and microclimate conditions.

In Song [64], the results of their study showed a positive correlation between airborne bacteria and temperature (*p* < 0.05); therefore, a high temperature creates a suitable environment for bacteria. In contrast, in less recent research, bacterial bioaerosol diversity was indicated to be significantly higher in winter [84]. Finally, a relative humidity (RH) above 80% seems to help with bacterial growth inhibition [64,85,86].

PM_2.5_ and ventilation have a seasonal influence on airborne contaminants. Air velocity can play a minor role in winter (when it is quite low), contributing to the ARGs increase, while in summer, the indoor speed velocity can push the spread of bioaerosol outside the buildings, constituting further risks for farmers and surrounding areas [64]. In winter, the absence of ventilation can contribute to the increase of NH_3_, dust, and pathogen concentrations in swine barns, increasing risks for pigs’ health [87].

Kumari [88] investigated fungi diversity and abundance in swine houses, finding that fungal OTU (operational taxonomic unit) composition is strongly related to RH, temperature, PM, NH_3_, and stocking density.

The community composition is wider and more heterogeneous in summer, when the major component is Ascomycota, thanks to its small dimensions, which make it more aerosolisable.

In addition, allergens related to fungi bound to PM_2.5_ are characterized by great variability among different structures. The abundance of these allergens depends on fungi genera, and it has been estimated to be around 13–25%. The most dangerous toxin is related to the Fusarium genera, which can cause infections in both animals and humans [88,89].

Each farm has its own dust composition depending on the breeding barn, farm buildings, management, and obviously the microclimatic conditions [83].

Since in recent decades limited importance has been given to the concentration of dust in livestock farms, it is necessary to introduce the habit of characterizing dust, and by deepening this aspect, it will be possible to ensure greater biosecurity and better animal and worker welfare.

## 5. Measurement Tips

To quantify the amount of dangerous gas, ClassyFarm indications are as follows: “The gas levels must be measured on farm using a portable gas detector to be placed in the middle of the box at the height of the animals (minimum 3 measurements, divided by type of box in different buildings or rooms). The measurement must be carried out at the head of the pig of the category considered in the assessment and in any case not exceeding one meter above the ground”.

In our experience, it is often necessary to be more thorough and carry out more measurements at different heights and at different points of the room, in order to avoid high levels of uncertainty and error, in particular in mechanical ventilation systems.

Obviously, on farms, air pollutants vary constantly. This depends on the ventilation system (natural or mechanical), the facility design, such as floor type (concrete floor, partial or total slatted floor), the under-floor pit, manure management, animal characteristics, time of day, and season [90]. Thus, continuous measurements represent the best technique to obtain reliable data, considering the high variations that temperature, humidity, and air pollutants can be subjected to during the day and among the different seasons [91]. Currently available technologies, budgets, and materials allow for high-quality measurements. However, proper planning, material selection, and regular maintenance reduce the risks of uncertainty [92].

### 5.1. The Importance of the Ventilation Rate Measurement

**Figure 2:**
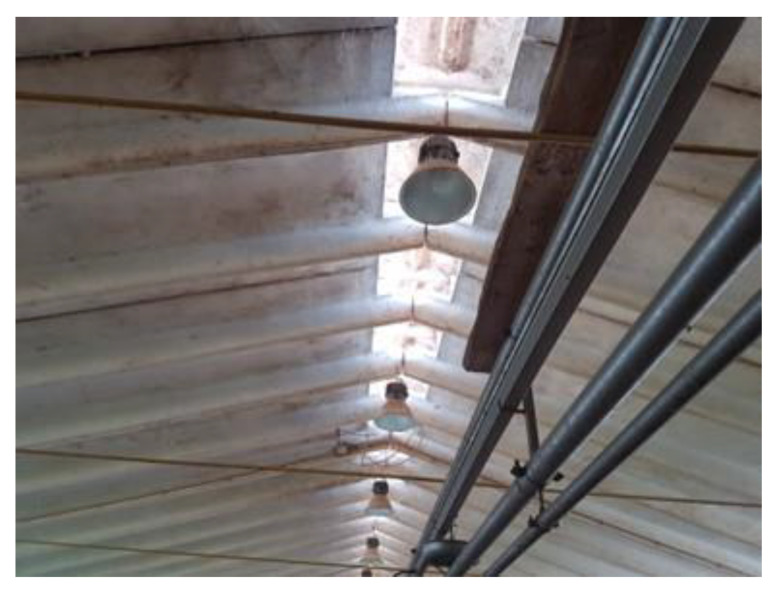
In a natural ventilation system, the opening in the roof ensures air exchange in a piggery.

**Figure 3:**
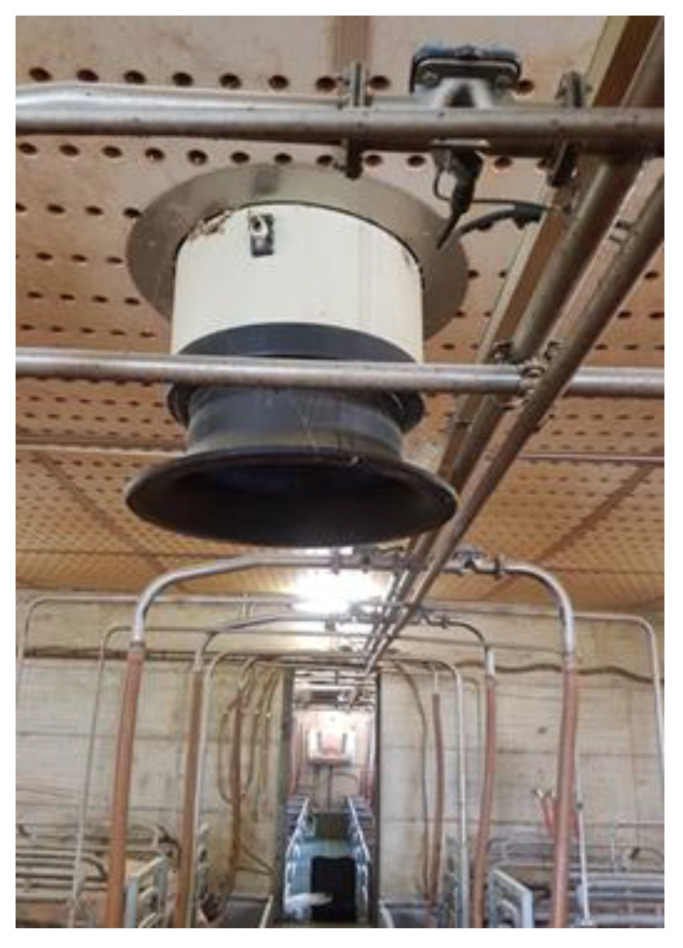
A mechanical fan guarantees the air exchange in an accurate way, according to thermal animal requirements.

**Figure 4:**
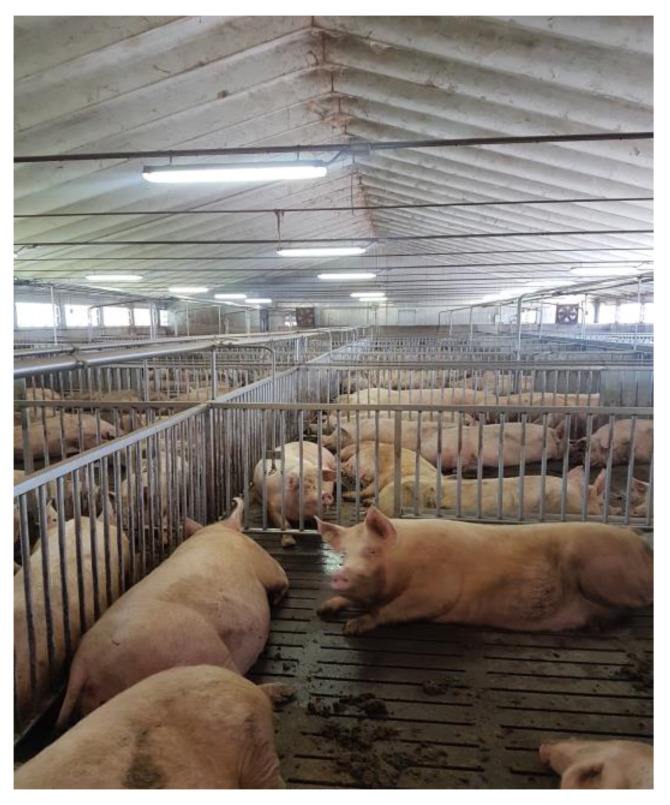
A mixed ventilation system in a piggery, characterized by natural ventilation in winter and mechanical fans in summer.

Ventilation, or the exchange of air volumes from an animal building to guarantee a good level of air quality and microclimatic parameters, affects the concentration of pollutants in animal confinements. It can be a system working under pressure or vacuum, and it can be distinguished in:

− Natural ventilation (Figure 2) is based on the exchange of air between the outside and the inside of a building without using mechanical fans.
− Mechanical ventilation (Figure 3), where facilities are equipped with fans that can be controlled by temperature sensors in order to meet animal thermal requirements, is an example reported in Table 2.

− Mixed systems, characterized by the use of natural ventilation in winter that is aided by mechanical fans in summer (see Figure 4).

Ventilation rate, which is the indicator of good air quality and temperature/humidity in animal confinements, can be measured using different instrumentation with different costs and different errors, as reported in Table 7.

Since pollutants distribution is strongly influenced by ventilation rate, it is important to determine its value, too. This can be conducted through different methods based on the type of ventilation (natural or mechanical), the availability of exhaust for physical measurements, the level of ventilation speed, and the required accuracy. Some methods are indirect (tracer gas, heat balance), while others are direct (fan anemometer, specialized instruments). Anemometers can be used to measure the speed of the air entering the building through doors, windows, or inlet surfaces to estimate the air volumes exchanged.

As reported by Costa [101,102] and Heber [92], gas emission rates from animal houses are based on ventilation rates and inlet and outlet concentrations of gases through the equation (Equation (2)):

(2) $$\mathrm{Ei}=\mathrm{Vi}\times \left(C,\;\mathrm{exhaust}-C,\;\mathrm{inlet}\right)$$

where:

Ei: emission of the pollutant at time i
Vi: Ventilation rate at time i
C, exhaust is the gas (i) concentration in the ventilation outlet
C, inlet is the gas (i) concentration in the ventilation inlet.

Since the error of the pollutant emission factor (dE) is limited by the sum of the errors of the pollutant’s concentration measurement (dC) and the ventilation rate measurement (dV), Equation (3) is:

(3) δE=δC+δV

Appropriate measuring methods are available to measure emissions in mechanically ventilated buildings, while in naturally ventilated buildings it is common to make more errors [103].

### 5.2. Pollutant Concentration Measurements: Gasses

Each pollutant needs to be investigated with proper techniques in order to define reference measurement methods to quantify and characterize them.

Different techniques and instruments can give different outputs, depending on the characteristics of the device used in terms of accuracy and detection limits.

Calvet [103] reported that there are three main types of errors that can compromise the accuracy of the measurement: random, systematic, and spurious errors. The first one can derive from an unexpected variation in the measured object. The second one represents the accuracy of the replicated measurements, and the last error type refers to instrument malfunction or human failure.

An adequate stabilization period and a proper sampling strategy can minimize recording disturbances.

The level of uncertainty associated with these devices is determined by the number of measurement positions, the location of sample collection, and the method used to analyze the data. Since the concentration of pollutants may fluctuate during the day or vary from day to day, depending on the different activity levels of animals [101,102], the average concentration over a period of 24 h should be determined by nearly continuous measurements for at least one week. So, the spatial variation inside the swine building is the main aspect to consider [104].

**Figure 5:**
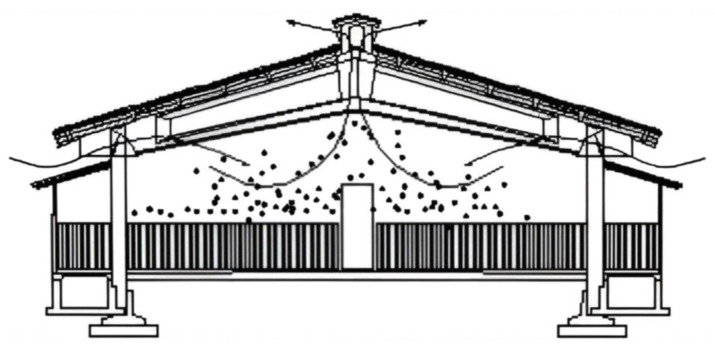
Ventilation rate (continuous lines) affects pollutants (dots) distribution and concentration.

Figure 5 shows the influence of ventilation rate on particle distribution in a piggery, a factor that can affect the measurements, with different concentrations detected during sampling activities.

Usually, in swine facilities, physical or chemical techniques are used to measure the concentration of noxious gases in animal houses [104]. Different available methods are reported in Table 8 [60].

The most used techniques are the optical ones, whose main advantage is the possibility of tracking concentration dynamics in real-time, so monitoring multiple gases with different concentrations at the same time [101,102,104].

### 5.3. Pollutant Concentration Measurements: Particulate Matter

Many reviews are available with regard to dust sampling techniques, for example the ones by Donham [67], Cambra-López [105], and Hamon [60]. Aerosols can be measured in two basic ways: by gravimetric technique, collecting them on a substrate as a PTFE filter, and simultaneously in real time, mainly by light scattering techniques [101,102].

The traditional gravimetric way involves collecting particles and then taking measurements over time, but the downside is that the particles may be modified by transport and collection processes. On the other hand, real-time techniques provide much quicker measurements, but they may only detect a limited degree of particle characterization [106]. Using them both can allow for more accurate results. This combined technique allows for the adjustment of the concentration of dust according to its specific weight since many dust counters are calibrated for different kinds of particulate matter. For example, the HAZ DUST, a particulate matter counter produced by SKC (863 Valley View Road, Eighty Four, PA), is calibrated using the Arizona dust, so an adjustment on the real-time counting must be performed, comparing the dust deposited on its filter and the real-time measurements [101,102].

## 6. Conclusions

The aim of this document is to stress the importance of assessing air quality in farms in order to suggest a more effective model for investigating welfare conditions in pig farming.

The main harmful effects caused by pollutants in pig farming were studied through the literature, which confirms the strong toxicity caused by these pollutants, emphasizing how the interaction between these factors, for example, dust and ammonia, can cause more pronounced damage in animals.

What can be deduced from this work is certainly the need for greater knowledge of the composition of farm dusts, as they contain and spread biological agents that can pose a danger to both animals and people.

It is important to remember that gaseous pollutants and bioaerosol in farms also result in a serious problem for the surrounding area and have a hazardous effect on residents’ health [91]. Proper evaluation of in-farm air quality helps to refine animal welfare conditions and helps to determine the emission of bioaerosols and gases into the surrounding area, too.

## Figures and Tables

**Table 1:** Optimal temperature requirements in pigs, indicated in the ClassyFarm system [12].

Weight	Temperature Range
5–14 kg	24–29 °C
14–23 kg	21–27 °C
23–34 kg	16–21 °C
34–82 kg	13–21 °C
>82 kg	10–21 °C

**Table 2:** Lower critical temperatures for rearing pigs at different weights and under different housing conditions [13].

	Weight (kg)		
	20	60	100
	Temperature (°C)		
SUMMER			
Draught-free insulated shelter	14	12	7
Draught-free shelter	17	15	11
Insulated shelter with straw bedding	10	8	2
WINTER			
Ventilated insulated shelter	18	16	13
Ventilated shelter	23	20	17
Non-insulated shelter with concrete floor	29	25	22

**Table 3:** Maximum acceptable concentration of pollutants inside pig buildings, according to Classyfarm indications.

Harmful Gas	Acceptable Concentration
NH_3_ (Ammonia)	10 ppm
CO_2_ (Carbon Dioxide)	3000 ppm
H_2_S (Hydrogen Sulfide)	0.5 ppm (5 ppm during evacuation)

**Table 4:** Negative effects on animal health modified by [8].

Pollutant	Effect
Ammonia (NH_3_)	Eye and/or respiratory tract inflammation, anorexia, irritation of the mucous membranes of the respiratory tract, reduction of immunity, and specific illnesses.
Carbon Dioxide (CO_2_)	Increased respiratory rate, breathing difficulties, possible light-headedness, dizziness, and unconsciousness.
Hydrogen Sulphide (H_2_S)	Eye and/or respiratory system inflammation, smell disturbances, anorexia, nausea, and diarrhoea.
Dust	Irritation of the respiratory and ocular system, the respirable fraction (<5 µm) can combine with harmful pollutants and originate the secondary particulate matter.

**Table 6:** Techniques to measure particulate matter in livestock buildings [60,68,69,70].

Analysis Classification	Substrate	Techniques	
All	Collect dust for chemical, physical, or biochemical evaluations	Electrostatic precipitation	Gravimetry Tapered element oscillating microbalance (TEOM) Beta-ray attenuation principle Photometry
Physical	MASS SAMPLING Concentration of particles (number and mass)	Total dust	
		Particle separation (Filtering by size)	Cyclone separator Cascade impactor Vertical elutriator
	PARTICLE COUNTING Particle-size distribution (PSD) (number and mass)	Light scattering	
		Beta attenuation Laser	
		Laser light microscopy	
		Scanning electron microscopy	
Chemical	Source apportionment	Scanning electron microscopy with X-ray (SEM-EDX)	
		Inorganic compound	X-ray fluorescence
			proton induced X-ray emission
		Organic compound	atomic absorption spectrophotometry inductively coupled plasma with atomic emission spectroscopy inductively coupled plasma with mass spectroscopy
	Toxins	Standard analytical methods (i.e., Limulus Amebocyte Lysate assay)	Endotoxin, aflatoxin,
	Allergens	Standard analytical methods	Protein analysis Antigenic analysis
Microbiological	Number of viable and non-viable bacteria/viruses/fungi	Andersen microbial sampler (AMS)	
		Nuclepore filtration and elution method (NFE)	
		all-glass impinger method (AGI)	

**Table 7:** Most commonly used techniques to measure the ventilation rate.

Ventilation Rate Measuring Technique	Error	Reference
Impeller anemometer	2–25%	Novalynx Corporation, Davis Instruments, [93]
Hot wire anemometer	0.5–25%	[94,95]
CO_2_ balance	15–40%	[96,97]
Thermic balance	30–100%	[28,96]
Tracer gas	10–50%	[41,98,99]
Ventilation rate control through a free running impeller	3%	[100,101,102]

**Table 8:** Techniques used to measure the concentration of noxious gases in animal houses.

	Equipment	Utility	Compounds	Accuracy	Detection Limit
Chemical Technique	Reactant colorimetric Tubes	approximative measurements	all compounds	85–98%	depends on analyzed compounds
Chemical + Physical Technique	Acid traps + Colorimetry	-	all compounds soluble in aqueous solutions	all compounds soluble in aqueous solutions	depends on analyzed compounds
Physical Technique	Gas Chromatography	pseudocontinuous measurements	depends on detectors	depends on detectors	depends on analyzed compounds
Optical Technique	Infrared photoacoustic detector	continuous measurements	all compounds	all compounds	~0.01–0.02 ppmv
Optical Technique	Fourier transform infrared (FTIR) Spectroscopy	-	NH_3_, CH_4_, CO_2_, N_2_O	NH_3_, CH_4_, CO_2_, N_2_O	~10 ppbv
Chemical Technique	Proton Transfer Reaction-Mass Spectrometry	continuous & real-time measurements	molecules with affinity to protons	molecules with affinity to protons	~1 ppbv
Physical Technique	Chemiluminescence NO Analyzer	-	NOx and NH_3_	NOx and NH_3_	~0.2 ppmv

## Data Availability

Not applicable.
